# Outcomes after stereotactic body radiotherapy for lung tumors, with emphasis on comparison of primary lung cancer and metastatic lung tumors

**DOI:** 10.1186/1471-2407-14-464

**Published:** 2014-06-23

**Authors:** Takaya Yamamoto, Keiichi Jingu, Yuko Shirata, Masashi Koto, Haruo Matsushita, Toshiyuki Sugawara, Masaki Kubozono, Rei Umezawa, Keiko Abe, Noriyuki Kadoya, Youjirou Ishikawa, Maiko Kozumi, Noriyoshi Takahashi, Ken Takeda, Yoshihiro Takai

**Affiliations:** 1 Department of Radiation Oncology, Tohoku University School of Medicine, Sendai, Japan; 2 Research Center for Charged Particle Therapy, National Institute of Radiological Sciences, Chiba, Japan; 3 Department of Radiological Technology, School of Health Sciences, Faculty of Medicine, Tohoku University, Sendai, Japan; 4 Department of Radiation Oncology, Hirosaki University School of Medicine, Aomori, Japan

**Keywords:** Stereotactic radiotherapy, SBRT, Primary lung cancer, Metastatic lung tumor, Oligometastasis, Prognostic factor

## Abstract

**Background:**

The goal of this study was to determine the prognostic factors associated with an improved overall outcome after stereotactic body radiotherapy (SBRT) for primary lung cancer and metastatic lung tumors.

**Methods:**

A total of 229 lung tumors in 201 patients were included in the study. SBRT of 45 Gy in 3 fractions, 48 Gy in 4 fractions, 60 Gy in 8 fractions or 60 Gy in 15 fractions was typically used to treat 172 primary lungs cancer in 164 patients and 57 metastatic lung tumors in 37 patients between January 2001 and December 2011. Prognostic factors for local control (LC) and overall survival (OS) were analyzed using a Cox proportional hazards model.

**Results:**

The median biologically effective dose was 105.6 Gy based on alpha/beta = 10 (BED10). The median follow-up period was 41.9 months. The 3-year LC and OS rates were 72.5% and 60.9%, and the 5-year LC and OS rates were 67.8% and 38.1%, respectively. Radiation pneumonitis of grades 2, 3 and 5 occurred in 22 petients, 6 patients and 1 patient, respectively. Multivariate analyses revealed that tumor origin (primary lung cancer or metastatic lung tumor, p < 0.001), tumor diameter (p = 0.005), BED10 (p = 0.029) and date of treatment (p = 0.011) were significant independent predictors for LC and that gender (p = 0.012), tumor origin (p = 0.001) and tumor diameter (p < 0.001) were significant independent predictors for OS.

**Conclusions:**

SBRT resulted in good LC and tolerable treatment-related toxicities. Tumor origin and tumor diameter are significant independent predictors for both overall survival and local control.

## Background

Stereotactic body radiotherapy (SBRT) results in a high local control rate for relatively small lung tumors and has low treatment-related toxicity, and it thus has many benefits for patients, especially patients who cannot undergo surgery [1-3]. SBRT may also be beneficial for patients who choose not to undergo surgery. For example, elderly patients, even elderly patients with no or only a few comorbidities and moderate lung function, often hesitate to undergo surgery because of concerns about postoperative complications, decline in activities of daily living, and progression of dementia, even in short-term admission. A time-trend analysis in the Netherlands showed that the number of elderly patients treated with radiotherapy for stage I non-small-cell lung cancer (NSCLC) has been increasing over time. This has occurred in parallel with increased availability of SBRT and has led to a decrease in untreated patients and no increase in surgically treated patients, despite operative and perioperative advances such as video-assisted thoracic surgery [4]. Furthermore, the outcome for stage I NSCLC treated with SBRT is now close to that after lobectomy, based on the results of a recent propensity-score matched analysis [5].

Retrospective analyses and one prospective analysis of SBRT for oligometastatic lung tumors have shown 2- to 3-year survival rates with good local control (LC) that compare favorably with surgical results [2,3]. If SBRT has an efficacy comparable to that of tumor resection, this technique may provide patients with a better quality of life, shorter time away from work, and minimal interruption of other treatment. This third issue may be a major advantage because systemic chemotherapy is often performed for targeting oligometastatic lung tumors, treating another lesion, or as maintenance or consolidation therapy for potential metastases.

Recently, the new notion of oligo-recurrence was proposed because the initial concept of oligometastases did not eliminate the uncontrolled primary site with several distant metastases. Oligo-recurrence has been suggested as a state of metachronous limited recurrence or metastases possibly cured with local therapy [6]. SBRT for lung oligo-recurrence with good LC rate and survival rate has also been reported [7].

Many prognostic factors for LC after SBRT have been reported, including tumor diameter, standardized uptake value (SUV) on [18F]fluorodeoxyglucose positron emission tomography (FDG-PET), low dose distribution, metastatic lung tumors, and colorectal lung metastases [8-14]. In contrast, there have been only a few studies in which prognostic factors for overall survival (OS) were examined, though clarification of such factors is important to maximize the benefit/toxicity ratio [14-17]. In this study, we retrospectively reviewed our results for lung tumors treated with SBRT with the goal of identifying prognostic factors associated with LC and OS and thus establishing a strategy for balancing the benefits and risks in use of SBRT.

## Methods

### Patients

A review of our institutional clinical database identified 215 patients who were treated with SBRT at our institute between January 2001 and December 2011. Patients with follow-up of < 3 months were excluded, leaving a total of 229 lung tumors in 201 patients that were analyzed retrospectively. Of these, 172 tumors in 164 patients were stage I primary lung cancer and 57 tumors in 37 patients were metastatic tumors. The main clinical and pathological pretreatment features are summarized in Tables 1 and 2. All numerical data are expressed as medians. The histological diagnoses of the 172 cases of primary lung cancer were adenocarcinoma (56 tumors), squamous cell carcinoma (40 tumors), large cell carcinoma (10 tumors), small cell carcinoma (2 tumors), NSCLC not otherwise specified (5 tumors), and bronchioloalveolar carcinoma (2 tumors), and 57 tumors were pathologically unproven. A metastatic lung tumor was defined as the appearance of a solid tumor during follow-up after treatment for the primary lesion. Inclusion criteria of metastatic tumors were that tumor diameter was 5 cm or less, both radiation oncologists and the physician assessed that SBRT of metastatic tumors would yield improved systemic control and prolonged survival, and consequently, all of the primary sites were controlled. Thirty-five cases were surgically resected, one case was esophageal cancer controlled by chemoradiation and another case was hepatocellular carcinoma controlled by transarterial chemoembolization. Two cases were synchronous metastases with a primary lesion, and 6 cases received additional chemotherapy. Biopsy for a metastatic lung tumor was performed only during follow-up of double cancer. Of the 57 metastatic tumors, 29 were metastases from colorectal cancer and 28 were metastases from other malignancies.

**Table 1:** Baseline patient characteristics

	**Primary lung cancer**	**Metastatic lung tumor**	**Total**
Number	164 patients	37 patients	201 patients
Median age (y)	78 (range: 40–92)	63 (range: 25–85)	76 (range: 25–92)
Gender			
Female	39 (23%)	13 (35%)	52 (25%)
Male	125 (76%)	24 (64%)	149 (74%)
ECOG performance status			
0-1	142 (86%)	37 (100%)	179 (89%)
2-3	22 (13%)	0 (0%)	22 (10%)
History of other malignancies			
Yes	81 (49%)	6 (16%)	87 (43%)
No	83 (50%)	31 (83%)	114 (56%)
Pack-year smoking			Median 37.5 (range: 0–180)
Never	35 (21%)	10 (43%)	45 (24%)
< 37.5	35 (21%)	10 (43%)	45 (24%)
≥ 37.5	88 (55%)	5 (20%)	93 (50%)
FEV1, % of predicted			Median 92.5 (range: 25.1-203.6)
< 92	54 (47%)	4 (66%)	58 (48%)
≥ 92	60 (52%)	2 (33%)	62 (51%)
Home oxygen therapy	13	0	13 (6%)
Operability			
Operable	64 (39%)	20 (54%)	84 (41%)
Inoperable	100 (60%)	17 (45%)	117 (58%)

*Abbreviations:ECOG* Eastern cooperative oncology group, *FEV1* forced expiratory volume in 1 second.

**Table 2:** Baseline tumor and treatment characteristics

	**Primary lung cancer**	**Metastatic lung tumor**	**Total**
Number	172 tumors	57 tumors	229 tumors
Tumor diameter (cm)			2.2 (range: 0.9-4.7)
≤ 2.0	73 (42%)	27 (47%)	100 (43%)
2.1-3.0	75 (43%)	23 (40%)	98 (42%)
3.1-5.0	24 (13%)	7 (12%)	31 (13%)
Tumor appearance			
Mainly solid component	160 (93%)	57 (100%)	217 (94%)
Mainly ground-glass opacity	12 (6%)	0 (0%)	12 (5%)
SUVmax on staging FDG-PET			5.9 (range: 0.6-22.8)
< 5.9	53 (50%)	4 (57%)	57 (50%)
≥ 5.9	53 (50%)	3 (42%)	56 (50%)
Prescription dose			
BED10 ≤ 105 Gy	84 (48%)	12 (21%)	96 (41%)
BED10 > 105 Gy	88 (51%)	45 (78%)	133 (58%)
Date of treatment			
2001-2005	76 (44%)	39 (68%)	115 (50%)
2006-2011	96 (55%)	18 (31%)	114 (50%)

*Abbreviations:SUVmax* maximum standardized uptake value, *FDG-PET* [18F]fluorodeoxyglucose positron emission tomography, *BED10* biological effective dose calculated using α/β = 10.

### SBRT procedure

We previously reported details of our SBRT technique and follow-up studies in patients with primary lung cancer [18]. Each patient was immobilized in a body frame (Vac-loc, Med-tek) and a clinician observed the respiratory tumor motion on a simulator (Ximatron, Varian Medical Systems). If breathing motion had a large effect on the tumor location, the clinician made the decision of whether to use an abdominal pressure belt, taking comorbidity and performance status into account. Treatment planning was then performed in the same position, using slow-rotation serial CT scanning (slice thickness, 2.5 mm; 4 s/slice). The gross tumor volume was defined as the visible extent of the tumor on the CT image in the lung window. The internal target volume (ITV) was determined from the slow-rotation CT images and from respiratory tumor motion on the simulator. The planning target volume was defined as the ITV with a 5-mm margin for set-up uncertainty. The SBRT plan was created with a 3D radiotherapy planning system (CADPlan/Eclipse, Varian Medical Systems). SBRT was delivered with a linear accelerator (Clinac 23EX, Varian Medical Systems) using 6 MV X-ray beams with 5 to 7 non-coplanar multistatic ports and/or multidynamic arcs.

Before June 2009, the dose calculation algorithm was based on the pencil beam method with heterogeneity correction (modified Batho power law), and 45 Gy in 3 fractions, 48 Gy in 4 fractions, 60 Gy in 8 fractions, or 60 Gy in 15 fractions to the isocenter was prescribed. In February 2004, we changed the prescription dose from 45 Gy in 3 fractions to 48 Gy in 4 fractions to unify the dose for the Japan clinical trial series. After June 2009, the dose calculation algorithm was changed to an analytical anisotropic algorithm and 40 Gy in 4 fractions or 50 Gy in 8 fractions covering 95% of the PTV (D95) was delivered. The median isocenter doses recalculated with pencil beam convolution were 46.5 Gy (range: 43.2-50.7 Gy) in 4 fractions and 56.2 Gy (range: 55.3-61.7 Gy) in 8 fractions. The choice of dose depended on tumor location and performance status: 60 Gy in 8 fractions, 60 Gy in 15 fractions or 50 Gy in 8 fractions was selected if the lung tumor was adjacent to critical structures such as the main bronchus, heart and great vessels, esophagus or stomach. Dose escalation was not performed for larger tumors or metastatic tumors. This study was approved by the ethical committee of Tohoku University Hospital and informed consent was obtained from all patients.

### Follow-up after SBRT

A clinical examination by a radiation oncologist and CT scanning were performed 4–6 weeks after SBRT to assess the pulmonary response. Thereafter, patients underwent follow-up examinations every 3–6 months for 2 years and then every 6 months after 2 years. Patients also underwent examinations by doctors in charge of the primary disease. Local recurrence or local failure was defined as local progression to ≥ 1.5 times the dimensions of the original tumor [18]. FDG-PET was sometimes performed to distinguish local recurrence from dense consolidation. The final diagnosis of local recurrence was made by physicians and radiation oncologists.

### Statistical analysis

Time to an event was calculated from the first day of SBRT to the day an event was confirmed. Statistical analyses were performed using JMP v. 10.0.2 (SAS Institute). Cumulative LC and OS rates were calculated using Kaplan-Meier curves, and a log-rank test was used to compare the curves. To analyze prognostic factors, univariate and multivariate analyses were performed using a Cox proportional hazards model. In these analyses, continuous variables that included missing values, such as SUVmax and pack-years smoking, were discretely divided at the sample median and then analyzed as categorical variables. Correlation coefficients for all variables were calculated to avoid multicollinearity. P < 0.05 was defined as significant in all tests. For analysis of various dosing schedules, the biological effective dose (BED10) was calculated using the following formula: BED10 = nd [1 + d/(α/β)], where n is the number of fractions, d is the isocenter dose per fraction, and α/β ratio is 10 Gy for the tumor. Toxicity was graded using the National Cancer Institute Common Terminology Criteria for Adverse Events ver. 4.0.

## Results

### Treatment results

**Figure 1:**
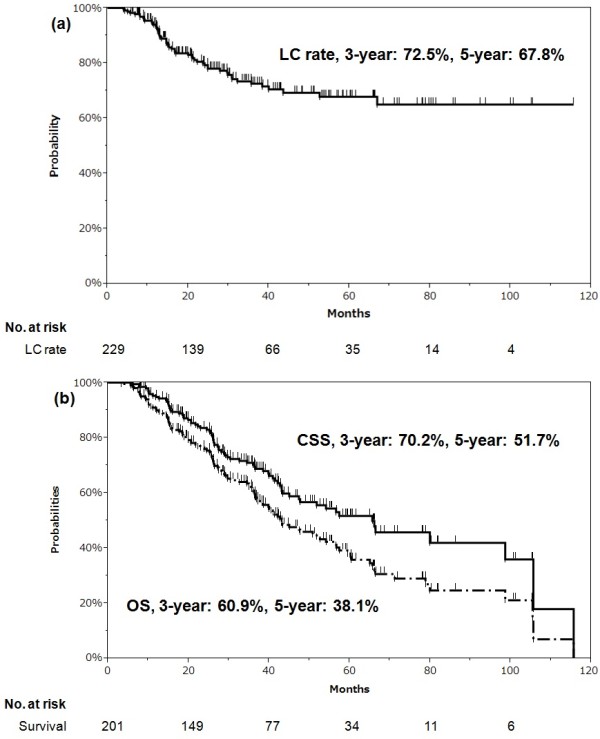
**Curves of (a) LC rate and (b) CSS and OS. (a)** Local control (LC) rate and **(b)** cause-specific survival (CSS) and overall survival (OS) in all patients in the study (Kaplan-Meier method).

**Figure 2:**
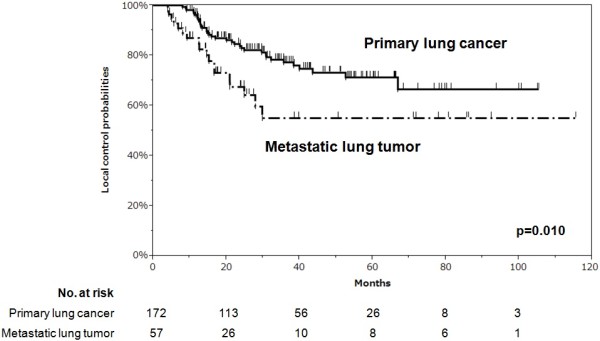
**Analysis comparing local control rate with primary lung cancer and metastatic lung tumors.** There was a significant difference in LC between primary lung cancer and metastatic lung tumors (p = 0.010, log-rank test).

The median follow-up period for all patients was 35.0 months (range: 3.3-115.6 months) and that for survivors was 41.9 months (range: 3.3-105.2 months). All of the primary sites with metastatic lung tumors were controlled at the time SBRT was started. During follow-up, local recurrence was observed in 54 of the 229 tumors. The median time to local failure was 14.9 months (range: 4.0-66.9 months). None the 12 tumors with mainly ground-glass components showed local failure; therefore, we had to exclude this factor from the analysis. Of the 201 patients, 77 died of the primary disease and 38 died of other causes. The median survival period was 43.1 months and the median cause-specific survival period was 65.6 months. The 3-year LC, cause-specific survival (CSS) and OS rates were 72.5% (95% confidence interval [CI]: 65.3-78.8), 70.2% (95% CI: 62.8-76.6) and 60.9% (95% CI: 53.6-67.7), respectively. The respective 5-year rates were 67.8% (95% CI: 59.6-75.1), 51.7% (95% CI: 42.8-60.5) and 38.1% (95% CI: 30.3-46.5) (Figure 1). There was a significant difference in LC between the primary lung cancer curve and metastatic lung tumors curve (p = 0.010, log-rank test) (Figure 2).

Radiation pneumonitis of grades 2 and 3 occurred in 22 and 6 patients, respectively. Steroids were administered to 14 patients. Grade 5 radiation pneumonitis occurred in one patient who had pathologically proven interstitial pneumonia before SBRT. The interstitial pneumonia was exacerbated after SBRT and then fatal bacterial pneumonia were involved in.

### Univariate and multivariate analyses

The results of univariate and multivariate analyses for LC and OS are shown in Tables 3 and 4. In univariate analysis, tumor origin (primary lung cancer vs. metastatic lung tumors; p = 0.017, hazard ratio [HR]: 0.48, 95% CI: 0.27-0.87), tumor diameter (per 1 cm increase; p < 0.001, HR: 1.87, 95% CI: 1.35-2.57), SUVmax (≥ 5.9 vs. < 5.9; p = 0.042, HR: 2.21, 95% CI: 1.02-5.03), date of treatment (2001–2005 vs. 2006–2011; p = 0.019, HR: 1.91, 95% CI: 1.11-3.39) and prescription dose (BED10: > 105 Gy vs. ≤ 105 Gy; p = 0.038, HR: 0.56, 95% CI: 0.32-0.96) were significant predictors for LC; and gender (female vs. male; p = 0.005, HR: 0.53, 95% CI: 0.33-0.83), tumor origin (primary lung cancer vs. metastatic lung tumors; p = 0.017, HR: 0.48, 95% CI: 0.27-0.87), tumor diameter (per 1 cm increase; p < 0.001, HR: 1.70, 95% CI: 1.33-2.16) and SUVmax (≥ 5.9 vs. < 5.9; p = 0.004, HR: 2.12, 95% CI: 1.26-3.64) were significant predictors for OS. In multivariate analysis, tumor origin (primary lung cancer vs. metastatic lung tumors; p < 0.001, HR: 0.21, 95% CI: 0.09-0.47), tumor diameter (per 1 cm increase; p = 0.005, HR: 1.70, 95% CI: 1.17-2.45) and prescription dose (BED10: > 105 Gy vs. ≤ 105 Gy; p = 0.029, HR: 0.51, 95% CI: 0.27-0.93) were significant independent predictors for LC; and gender (female vs. male; p = 0.012, HR: 0.56, 95% CI: 0.34-0.88), tumor origin (primary lung cancer vs. metastatic lung tumors; p = 0.001, HR: 0.45, 95% CI: 0.28-0.73) and tumor diameter (per 1 cm increase; p < 0.001, HR: 1.70, 95% CI: 1.30-2.21) were significant independent predictors for OS.

**Table 3:** Univariate analysis for local control (LC) and overall survival (OS)

**Variables**	**UVA for LC**		**UVA for OS**	
	**HR (95% CI)**	**P value**	**HR (95% CI)**	**P value**
Age (per 10-y increase)	0.95 (0.80-1.15)	0.617	0.93 (0.80-1.11)	0.428
Gender (female vs. male)	0.66 (0.34-1.19)	0.177	0.53 (0.33-0.83)	0.005*
Performance status (0–1 vs. ≥ 2)	1.19 (0.52-3.43)	0.703	0.86 (0.50-1.58)	0.619
Tumor origin (primary lung cancer vs. metastatic lung tumors)	0.48 (0.27-0.87)	0.017*	0.53 (0.34-0.84)	0.007*
History of other malignancies (yes vs. no)	0.70 0.38-1.21)	0.212	0.83 (0.56-1.20)	0.331
Pack-year smoking (≥ 37.5 vs. < 37.5)	1.38 (0.77-2.51)	0.266	0.85 (0.57-1.26)	0.426
Operability (operable vs. inoperable)	0.68 (0.38-1.19)	0.185	0.76 (0.52-1.12)	0.173
FEV1, % of predicted (≥ 92% vs. < 92%)	1.01 (0.49-2.05)	0.972	0.84 (0.51-1.36)	0.494
Tumor diameter (per 1 cm increase)	1.87 (1.35-2.57)	<0.001*	1.70 (1.33-2.16)	<0.001*
SUVmax (≥ 5.9 vs. < 5.9)	2.21 (1.02-5.03)	0.042*	2.12 (1.26-3.64)	0.004*
Date of treatment (2001–2005 vs. 2006–2011)	1.91 (1.11-3.39)	0.019*	1.36 (0.93-1.99)	0.106
Prescription dose (BED10: > 105 Gy vs. ≤ 105 Gy)	0.56 (0.32-0.96)	0.038*	0.94 (0.65-1.37)	0.760
Radiation pneumonitis (≥ 2 vs. < 2)	0.85 (0.34-1.78)	0.689	0.72 (0.40-1.22)	0.225

*Abbreviations:UVA* univariate analysis, *LC* local control, OS overall survival, *HR* hazard ratio, *CI* confidence interval, *FEV1* forced expiratory volume in 1 second, *SUVmax* maximum standardized uptake value, *BED10* biological effective dose calculated using α/β = 10. *p < 0.05.

**Table 4:** Multivariate analysis for local control (LC) and overall survival (OS)

**Variables**	**MVA for LC**		**MVA for OS**	
	**HR (95% CI)**	**P value**	**HR (95% CI)**	**P value**
Gender (female vs. male)	n.s.		0.56 (0.34-0.88)	0.012*
Tumor origin (primary vs. metastatic lung tumors)	0.21 (0.09-0.47)	<0.001*	0.45 (0.28-0.73)	0.001*
Tumor diameter (per 1 cm increase)	1.70 (1.17-2.45)	0.005*	1.70 (1.30-2.21)	<0.001*
SUVmax (≥ 5.9 vs. < 5.9)	1.50 (0.66-3.61)	0.331	1.41 (0.81-2.48)	0.218
Prescription dose (BED10: > 105 Gy vs. ≤ 105 Gy)	0.51 (0.27-0.93)	0.029*	n.s	
Date of treatment (2001–2005 vs. 2006–2011)	2.22 (1.19-4.23)	0.011*	n.s	

*Abbreviations:MVA* multivariate analysis, *LC* local control, *OS* overall survival, *HR* hazard ratio, *CI* confidence interval, *SUVmax* maximum standardized uptake value, *BED10* biological effective dose calculated using α/β = 10; *n.s.* not significant. *p < 0.05.

### Subgroup analysis of metastasis from colorectal cancer

**Figure 3:**
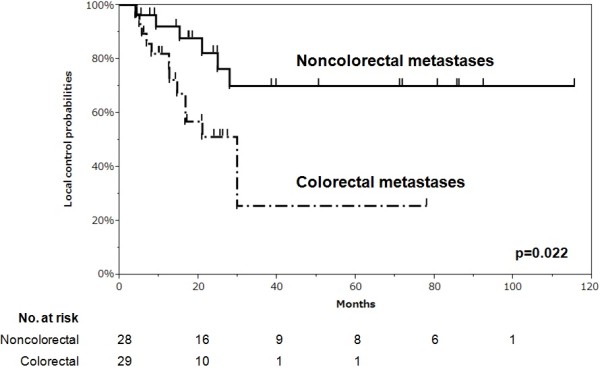
**Subanalysis comparing patients with metastases from colorectal and non-colorectal cancer.** There was a significant difference in local control between subgroups with metastases from colorectal cancer and metastases from noncolorectal disease (p = 0.022, log-rank test).

The results of a subanalysis comparing patients with metastases from colorectal cancer and non-colorectal cancer are shown in Figure 3. The difference in LC between the two subgroups was significant (p = 0.022).

## Discussion

This study was performed as a review of a single center experience of SBRT for lung tumors. In multivariate analysis, metastatic lung tumors, increased tumor size, early date of treatment and prescription dose of BED10 > 105 Gy were significant factors for unfavorable LC. LC itself was associated with OS in univariate analysis (HR: 2.02, 95% CI: 1.33-3.01, p = 0.001), indicating that a strategy for improving LC is important. For this purpose, dose escalation may be reasonable for metastatic lung tumors or larger tumors, given the relatively low toxicity in our patients, despite the poor pulmonary background and the presence of complications. However, this strategy may not be applicable in all cases and may not always give the expected outcome because critical structures can prevent delivery and distribution of a sufficient dose. This problem is also likely to be increased in dose escalation for larger tumors or multiple metastatic tumors.

Prescription dose of BED10, which was divided into two categories at the sample median (> 105 Gy, ≤ 105 Gy), was an independent predictor. The results confirmed previous findings [8,14]. Prescription dose ≤ 105 Gy was often used when the target was close to a critical structure. Thus, this strategy was relatively safe but might contribute to relatively low LC rate because of the low prescription dose.

The date of treatment was a significant factor for LC, possibly due to improvements in contouring (including spiculation of the GTV) and radiation planning (introduction of the concept of the homogeneity index). Furthermore, LC rates naturally became lower as the follow-up period became longer. The median follow-up periods in 2001–2005 and 2006–2011 were 50.2 months and 41.6 months, respectively, and this difference would also therefore affect the results.

We found that a metastatic lung tumor was an independent poor prognostic factor for LC and OS. For LC, this result confirms a previous finding of Hamamoto et al. [13] and is based on a much longer follow-up period in the current study.

The poor LC rate of a metastatic lung tumor may be the result of formation of a metastasis. Metastatic tumor cells are the particularly potent malignancy of tumor, which are the most aggressive cells in the neoplasm because most malignant cells entering the metastatic process are killed, especially in the blood circulatory system [19]. Also, in some cases, adjuvant chemotherapy or hormonal therapy is performed after surgery. Cells with high metastatic potential are resistant to this treatment based on genetic instability due to a several-fold increase in the rate of mutation compared to tumor cells with lower metastatic potential [20]. This genetic instability increases the angiogenic and invasive potential of tumor cells, and the tumor cells escape from immune surveillance, thus increasing the chance of metastasis. Through these steps, and after formation of a metastasis, tumors in advanced stages develop biological heterogeneity and increased radioresistance before and during SBRT.

Another reason for the poor LC rate of a metastatic lung tumor might be the state of the patient. The effects of disease treatment and disease progression may lead to cachexia and anemia temporarily or throughout the course of the disease, making the tumor more hypoxemic. Tumor hypoxemia has been hypothesized to lead to tumor growth and resistance to therapy because of induction of angiogenesis, genetic mutations, resistance to apoptosis, and resistance to free radicals from radiotherapy [21]. These changes may be one of the reasons for the poor LC of metastatic lung tumors, but both the “seed” aspect and the “soil” aspect may be important. According to the “seed and soil” hypothesis, metastasis is the product of interactions between selected cancer cells (the “seeds”) and specific organ microenvironments (the “soil”) [22]. In the lung environment, stromal products cause upregulation of P-glycoprotein in the cells, which prompts excretion of a variety of toxic compounds, including chemotherapeutic drugs, resulting in enhanced resistance to drugs [23]. Similarly, through tumor-stroma interactions, some stromal products may cause increased radioresistance of metastatic cells. If a microenvironment is changed to a rich soil due to interactions and molecular factors, circulating tumor cells may be reseeded, as at the primary site [24]. Therefore, modulation of a tumor microenvironment using approaches such as antiangiogenic therapy may be effective in some metastatic cases.

In subgroup analysis, metastases from colorectal cancer showed a lower local control curve than did metastases from non-colorectal cancer, consistent with previous findings [12]. However, we cannot conclude that colorectal metastases are more radioresistant than other metastases because of the small sample size and the variety of non-colorectal tumors, given that malignant diseases have various natural histories. For example, our cases included two cases of metastasis from thyroid cancer, which generally shows slow growth. Our findings do suggest that metastases from colorectal cancer are aggressive and are likely to cause short-term local relapse.

Although our inclusion criteria for metastatic lung tumors included both oligometastasis and oligo-recurrence, SBRT as a local therapy might be effective in both situations. According to the Norton-Simon hypothesis, the efficacy of chemotherapy is proportional to the growth rate of the tumor, and the growth is faster when the tumor is not bulky. Therefore, treatment of a bulky tumor with a slower growth rate and thus lower sensitivity to chemotherapy might make the remaining tumor cells more sensitive to chemotherapy [25,26]. Improvement in LC by reducing metastases should lead to increased OS for patients including both patients with oligometastasis and patients with oligo-recurrence.

Tumor diameter was identified as an independent factor for OS in multivariate analysis. This was probably due to the poor LC of a large tumor and the increased tendency for metastasis. In pulmonary resection with node dissection for clinical stage I NSCLC, 19.4% of the patients were found to have pathologically positive nodes, with a particularly high rate of 31.8% for unexpected positive nodes in clinical stage IB cases [27]. Thus, this analysis showed that tumor size and solid consistency were independent predictors for node metastasis but did not show that unexpected pathologically positive nodes affected OS. However, in our analysis of stage I NSCLC cases, clinical hilar lymph node or mediastinal lymph node failure was a significant factor for unfavorable OS (HR: 1.73, 95% CI: 1.04-2.78, p = 0.034). This finding suggests that staging must be performed thoroughly before SBRT. In an operable case, assessment of suspicious lymph nodes by endoscopic ultrasound-guided fine-needle aspiration may be necessary. Adjunct therapy for a larger tumor should also be considered in cases in which this is possible.

Female gender was also found to be a favorable factor for OS but not for LC. This may be because smoking was less common in females, and this may reduce smoking-related complications and development of another malignancy. Also, epidermal growth factor receptor-tyrosine kinase inhibitor (EGFR-TKI) therapy for primary lung cancer with EGFR mutation was more common in females, with 6 of 16 female patients (37%) receiving an EGFR-TKI in the course of treatment, in contrast to only 2 of 66 male patients (3%).

Analysis of tumor consistency was not included in the study because there was no local failure of ground-grass opacity (GGO) tumors and there was only one case in which disease progression occurred. Most tumors with mainly a GGO component were atypical adenomatous hyperplasia, adenocarcinoma in situ (formerly referred to as bronchioloalveolar carcinoma) or minimally invasive adenocarcinoma, all of which have a relatively good prognosis [28]. Thus, assessment of GGO tumors will require more cases and longer follow-up periods. However, it may be more useful to examine the safety of treating multiple sites by SBRT. Tumors with mainly a GGO component are often multifocal, and for all such tumors (not limited to GGO tumors), the lifetime incidence of a second primary tumor after surgical resection is > 10% [29]. More cases of multi-site treatment should emerge as the outcome of SBRT improves, and this will allow an evaluation of the safety of repetitive treatment with SBRT.

There were several limitations to this study. First, this study was a retrospective single institute analysis with a limited sample size, the number of metastatic lung tumors being particularly small. Second, various treatment protocols were included in the analysis. There were a variety of total doses, methods of dose prescription and fractionation schema.

## Conclusions

SBRT gave a high local control rate with tolerable treatment-related toxicities. Our results suggest that dose escalation should be considered for larger and/or metastatic lung tumors to improve the balance of benefits and risks of SBRT and to obtain better overall survival.

## Abbreviations

SBRT: Stereotactic body radiotherapy; LC: Local control; OS: Overall survival; NSCLC: Non-small-cell lung cancer; SUV: Standardized uptake value; FDG-PET: [18F]fluorodeoxyglucose positron emission tomography; BED: Biological effective dose; HR: Hazard ratio; EGFR-TKI: Epidermal growth factor receptor-tyrosine kinase inhibitor; ECOG: Eastern cooperative oncology group; FEV1: Forced expiratory volume; UVA: Univariate analysis; CI: Confidence interval; MVA: Multivariate analysis; n.s.: Not significant.

## Competing interests

The authors declare that they have no competing interests.

## Authors’ contributions

TY designed the analysis, reviewed the clinical data, performed statistical analysis and drafted the manuscript. KJ treated the patients, reviewed the clinical data and revised the manuscript. YS and MK treated the patients and reviewed the clinical data. HM, TS, MK, RU, KA, NK, YI, MK, NT and KT treated patients and collected clinical data. YT conceived the study, reviewed the clinical data, and treated patients. All authors read and approved the final manuscript.

## Pre-publication history

The pre-publication history for this paper can be accessed here:

http://www.biomedcentral.com/1471-2407/14/464/prepub
